# Zearalenone and Its Derivatives α-Zearalenol and β-Zearalenol Decontamination by *Saccharomyces cerevisiae* Strains Isolated from Bovine Forage

**DOI:** 10.3390/toxins7083297

**Published:** 2015-08-20

**Authors:** Luiz Keller, Luís Abrunhosa, Kelly Keller, Carlos Alberto Rosa, Lilia Cavaglieri, Armando Venâncio

**Affiliations:** 1 Departamento de Zootecnia e Desenvolvimento Agrossocioambiental Sustentável, Universidade Federal Fluminense (UFF), Rua Vital Brazil n.64, Niterói 24230-340, RJ, Brazil; E-Mail: kellers@bol.com.br; 2 CEB (Centre of Biological Engineering), University of Minho, Campus de Gualtar, Braga 4710-057, Portugal; E-Mail: avenan@deb.uminho.pt; 3 Departamento de Medicina Veterinária Preventiva, Universidade Federal de Minas Gerais, Escola de Veterinária, Belo Horizonte 31270-901, MG, Brazil; E-Mail: kelly.medvet@gmail.com; 4 Departamento de Microbiologia e Imunología Veterinária, Universidade Federal Rural do Rio de Janeiro, Instituto de Veterinária, Rodovia BR 465 Km 7, Seropédica 23890-000, RJ, Brazil; E-Mail: shalako1953@gmail.com; 5 Departamento de Microbiología e Inmunología, Universidad Nacional de Río Cuarto, Ruta 36 km. 601, Río Cuarto 5800, Córdoba, Argentina; E-Mail: lcavaglieri@exa.unrc.edu.ar

**Keywords:** ZEA, α-ZOL, β-ZOL, detoxification, adsorption, *Saccharomyces cerevisiae*

## Abstract

Zearalenone (ZEA) and its derivatives are mycotoxins with estrogenic effects on mammals. The biotransformation for ZEA in animals involves the formation of two major metabolites, α- and β-zearalenol (α-ZOL and β-ZOL), which are subsequently conjugated with glucuronic acid. The capability of *Saccharomyces cerevisiae* strains isolated from silage to eliminate ZEA and its derivatives α-ZOL and β-ZOL was investigated as, also, the mechanisms involved. Strains were grown on Yeast Extract-Peptone-Dextrose medium supplemented with the mycotoxins and their elimination from medium was quantified over time by HPLC-FL. A significant effect on the concentration of ZEA was observed, as all the tested strains were able to eliminate more than 90% of the mycotoxin from the culture medium in two days. The observed elimination was mainly due to ZEA biotransformation into β-ZOL (53%) and α-ZOL (8%) rather than to its adsorption to yeast cells walls. Further, the biotransformation of α-ZOL was not observed but a small amount of β-ZOL (6%) disappeared from culture medium. ZEA biotransformation by yeasts may not be regarded as a full detoxification process because both main end-products are still estrogenic. Nonetheless, it was observed that the biotransformation favors the formation of β-ZOL which is less estrogenic than ZEA and α-ZOL. This metabolic effect is only possible if active strains are used as feed additives and may play a role in the detoxification performance of products with viable *S. cerevisiae* cells.

## 1. Introduction

The growth of filamentous fungi on agricultural commodities determines their deterioration and quality loss, aside from their eventual contamination with mycotoxin. Mycotoxins are toxic substances for humans and animals through ingestion, contact, and inhalation, which are produced by fungal secondary metabolism. The major mycotoxins found in agricultural products are aflatoxins (AFs), zearalenone (ZEA), deoxynivalenol and its derivatives, fumonisins, patulin, and ochratoxin A (OTA). For livestock production, the presence of mycotoxins in feed determines economic losses derived from poor growth and feed conversion, increased mortality, carcass refuse and many other associated problems. In fact, numerous health syndromes in livestock are likely to result from the ingestion of mycotoxins present in spoiled silage or feed [1]. 

Zearalenone has relatively low acute toxicity, but it interferes strongly with the reproductive tract of animals. Among other effects, ZEA decreases animal fertility, induces fibrosis in the uterus, breast cancer, and endometrial carcinoma [2]. The biotransformation of ZEA in animals involves the formation of two major metabolites, α-zearalenol and β-zearalenol (α-ZOL and β-ZOL), which are subsequently conjugated with glucuronic acid. α-ZOL shows higher estrogenicity than ZEA, but β-ZOL is less estrogenic [3,4].

Pigs have been found to convert ZEA predominantly to α-ZOL in the liver, the small intestines, and even in granulose cells. This, together with the low glucuronidation capacity seen in most pig breeds, might explain the high sensitivity of pigs towards the endocrine effects of ZEA [3]. This hypothesis is also supported by the observed insensitivity of rats and cows to ZEA estrogenicity because they predominantly convert ZEA to β-ZOL, the least active ZEA metabolite [5].

Anti-mycotoxin additives (AMA) are defined as a group of products able to adsorb, inactivate, or neutralize mycotoxins in the gastrointestinal tract of animals. Some of them are based on yeast cell walls and use the natural structure of *Saccharomyces cerevisiae* cell walls to bind several mycotoxins such as AFs, OTA, and ZEA [6,7,8], reducing the mycotoxins gastrointestinal absorption. In addition, yeast based products may act as a probiotic contributing to improve general animal health because they stimulate their immune systems and promote the integrity of intestinal mucosa [9]. The composition of yeast cell walls determines their binding performance; mannan oligosaccharides (MOS) components of *S. cerevisiae* cell walls play the major role in mycotoxin binding [10]. Nonetheless, Yiannikouris *et al.* [11,12] proposed that the β-D-glucan portion of the yeast cell wall also interacts with mycotoxin molecules in a very favorable and stable association. Mannan esterified with glucan has also been found to have important binding characteristics. Aravind *et al.* [13] suggested that addition of dietary esterified glucomannan is effective in broilers to counteract *in vivo* toxic effects of feed naturally contaminated with AFs, OTA, ZEA, and T-2 toxin.

Currently, the adsorbent capacity displayed by the cell wall and whole cells of *S. cerevisiae* has become the focus of numerous *in vitro* and *in vivo* studies worldwide [10,14,15,16]. Their binding performances are strain-dependent as well as sensitive to *in vivo* conditions such as the variations of pH in animal’s gastrointestinal tract. Therefore, yeast-based AMA should be tested individually for its beneficial properties. Cell walls and viable whole cells are already being used as a prebiotic or probiotic for improving animal performance [6,17,18]. Probiotics and AMA, are today a real tool in modern livestock farming, proving that research in this subject offers improvements for the sustainable development of animal livestock.

The aim of this study was to determine the *in vitro* performance of three *S. cerevisiae* strains isolated from bovine forages for the detoxification of ZEA, α-ZOL, and β-ZOL. The adsorption and biodegradation capacities of each strain were evaluated. 

## 2. Results and Discussion

### 2.1. Adsorption of ZEA, α-ZOL, and β-ZOL by S. cerevisiae Strains

**Figure 1:**
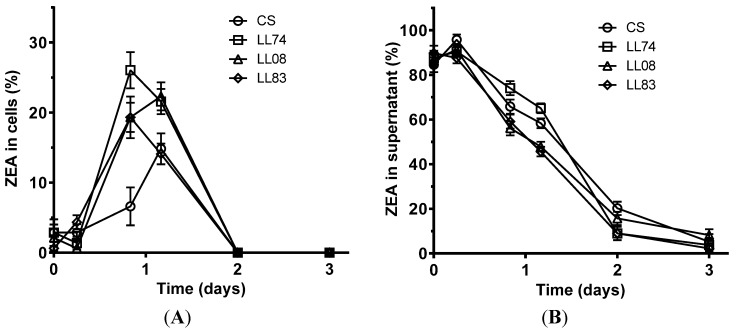
ZEA over time in cells and supernatants when they were extracted separately. (**A**) ZEA detected in cell fraction; (**B**) ZEA detected in liquid fraction. Values are expressed in percentage of initial amount of ZEA added to culture media. Data are means ± standard deviation of three repetitions.

Adsorption of ZEA to yeast cell walls was observed in the first 24 hours but, thereafter, adsorption declined and was no longer observed after 2 days of growth (Figure 1A). The maximum adsorption of ZEA was observed with strain LL74 and reached 26% of the initial concentration of ZEA. Under these conditions, the less active strain was CS, which only adsorbed about 15% of the total initial amount of the mycotoxin. These results indicate that yeasts cells can effectively adsorb ZEA.

In the supernatant, a decline in the amount of ZEA in solution was also observed (Figure 1B). Nonetheless, this decline continued after the first day of incubation, which may be explained by the co-occurrence of an adsorption and a reaction phenomenon. In the beginning of yeast cultivation, higher ZEA concentration in the supernatant has favoured its adsorption to cell walls, approaching the equilibrium state between bound and free ZEA. However, as ZEA concentration in the supernatant decreased due to its transformation by the yeast, a release of ZEA from the cell wall occurred (again to approach the equilibrium state between bound and free ZEA). These observations are indicative of a metabolization of ZEA by yeasts and further studies were conducted to evaluate the involved mechanisms. Comparing the adsorption capacity of the tested strains, a lower adsorption capability of the commercial strain was observed.

**Figure 2:**
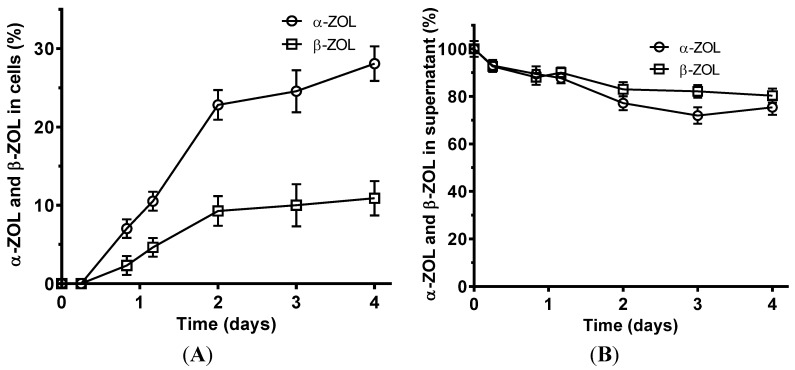
α-ZOL and β-ZOL over time in cells and supernatants when they were extracted separately. (**A**) α-ZOL and β-ZOL detected in cell fraction; (**B**) α-ZOL and β-ZOL detected in liquid fraction. Values are expressed in percentage of initial amount of α-ZOL and β-ZOL added to culture media. Data are means ± standard deviation of three repetitions.

To evaluate if yeasts were also able to adsorb the major ZEA metabolic products (α-ZOL and β-ZOL), experiments with Yeast Extract-Peptone-Dextrose broth (YPD) supplemented with those metabolites were also performed. The most effective yeast (strain LL74) was able to adsorb 28% of α-ZOL from culture media and about 11% of β-ZOL, after an incubation period of four days. α-ZOL and β-ZOL detected in cells and supernatants over time are presented in Figure 2.

It is known that adsorption of mycotoxins by yeast used as probiotic can be affected by the gastrointestinal environment. Therefore the adsorption of ZEA was also evaluated under simulated gastrointestinal environment. At pH 3, yeasts cells could adsorb between 31% and 43% of ZEA (Table 1). The adsorption at pH 3 was significantly greater than at pH 6, which ranged only from 13% to 24%. This is consistent with what is described in the literature, where more acidic conditions favor the adsorption on yeast cell walls [19]. Strain LL08 exhibited a significant higher adsorption at pH 3 and pH 6. The remaining strains showed no significant differences between them. 

**Table 1:** Adsorption of ZEA by *S. cerevisiae* strains in gastric and intestinal simulation experiments conducted at pH 3 and 6.

Strains	ZEA (µg/mL)	Yeast cells (mg/mL)	pH 3	pH 6
			Adsorption * (%)	Adsorption * (%)
Controls	2.1	0.0	---	---
CS	2.1	2.0	31.2 ± 2.3 ^a^	17.1 ± 1.8 ^a^
LL74	2.1	2.0	33.1 ± 1.5 ^a^	14.4 ± 2.5 ^a^
LL08	2.1	2.0	43.1 ± 3.1 ^b^	23.7 ± 1.6 ^b^
LL83	2.1	2.0	34.2 ± 2.6 ^a^	13.2 ± 1.0 ^a^

* Data are expressed as means ± standard deviation of three repetitions. ^a,b^ Data with different letters in each column are significantly different (*p* < 0.05).

A good performance at acidic and neutral conditions is a requirement for any anti-mycotoxin additive, which requires binding sites for adsorption during the passage through the all gastrointestinal tract. More interesting than the adsorption effect, presented by the AMA, is the positive effect of viable cells (probiotic) on animal health. The effect of viable *S. cerevisiae* is described below.

### 2.2. Biotransformation of ZEA, α-ZOL, and β-ZOL by S. cerevisiae Strains

**Figure 3:**
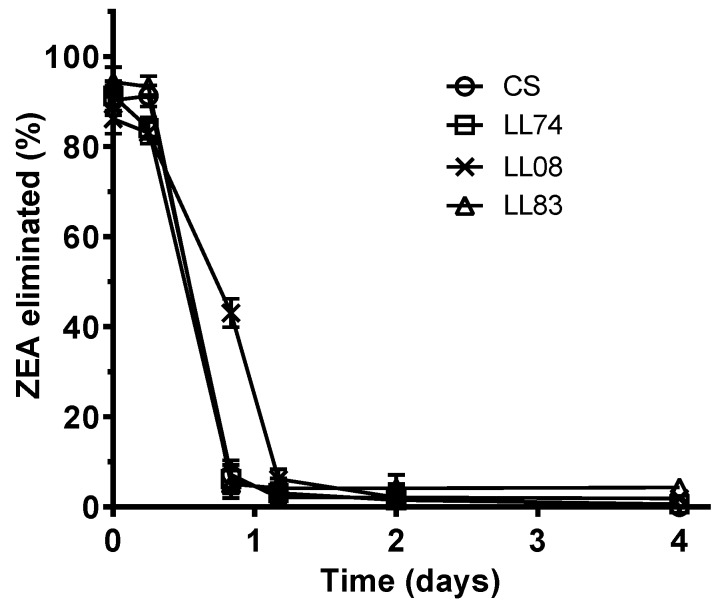
Elimination of ZEA by *S. cerevisiae* strains over time when cells and supernatants were extracted together. Values were expressed in percentage of initial amount of ZEA added to culture media. Data are means ± SD (standard deviation) of three repetitions.

The concentration of ZEA detected in culture broth after the first day of incubation is presented in Table 2. Its disappearance from culture broths and cells (extracted together) was observed for all the tested strains. ZEA elimination from culture media over time by tested yeasts is depicted in Figure 3. The CS, LL74, and LL83 strains were the fastest eliminating strains, achieving eliminations of 93%, 94%, 95%, respectively, during the first 20 h of incubation. Strain LL08 was the slowest one, exhibiting a significant lower transformation of ZEA.

The elimination capacity of strains showed as the time taken by strains to eliminate 50% and 90% of ZEA from culture media is also depicted in Table 2. The most active strains (*S. cerevisiae* CS, LL74 and LL83) were able to eliminate more than 90% of ZEA present in the culture media in one day (Figure 3). The time required by those strains to eliminate 50% and 90% of ZEA was estimated at 9 h and 18 h, respectively (Table 2). For the same level of elimination, the less active strain (*S. cerevisiae* LL08) required 17 and 28 h, respectively.

**Table 2:** ZEA concentration in culture broths after day 1 of incubation and time taken by *S. cerevisiae* strains to biotransform 50% and 90% of ZEA (DT50 and DT90, respectively).

Strains	ZEA * (μg/mL)	Logistic model		Curve Fitted Errors	
		DT50 (h)	DT90 (h)	*R*^2^	Sy.x (%)
Controls	1.95 ± 0.08	---	---	---	---
CS	0.04 ± 0.01 ^a^	9	18	0.9996	1.2
LL74	0.06 ± 0.02 ^a^	9	18	0.9995	1.3
LL08	0.12 ± 0.05 ^b^	17	28	0.9925	4.9
LL83	0.08 ± 0.02 ^a,b^	9	18	0.9961	3.8

* Data are expressed as means ± standard deviation of three repetitions. ^a,b^ Data with different letters are significantly different (*p* < 0.05)

**Figure 4:**
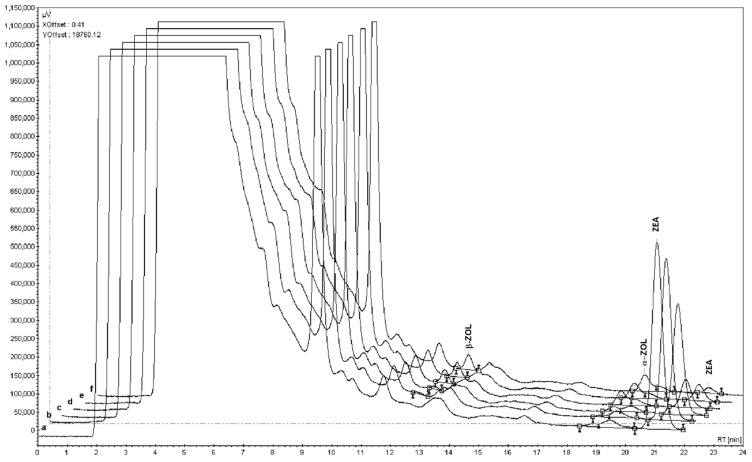
Chromatograms obtained for *S. cerevisiae* LL08 concerning ZEA and its derivatives α-ZOL and β-ZOL (*a* to *f*: 0, 6, 20, 28, 48 and 96 h).

An example of the chromatograms obtained for strain LL08 is depicted in Figure 4. The biotransformation effect was seen in all strains tested and, therefore, it is important to emphasize that strains isolated from silages metabolize ZEA in a similar way to the commercial strain (*S. cerevisiae*, CS). This is indeed important, because it demonstrates that the metabolization mechanism of ZEA is widespread to commercial and wild strains of *S. cerevisiae* and, therefore, wild strains may have potential for developing new anti-mycotoxin products.

The metabolization of ZEA into α-ZOL and β-ZOL was also investigated because it was observed before [20]. α-ZOL and β-ZOL were detected in chromatograms using commercial standards to identify the retention times (Figure 4). On average, at the end of the incubation period, ZEA represented about 4% of its initial amount, α-ZOL represented 8% of the initial amount of ZEA added to culture media and β-ZOL represented 53%, meaning that about one third of the ZEA added to culture media has been metabolized into other compounds that could not be identified with the current methodology. Also in a previous work, the fermentation of a wort-containing ZEA with a strain of *S. cerevisiae* resulted in a conversion of 69% of the mycotoxin into β-ZOL, a metabolite with lower estrogenic activity than the parent compound [21]. Despite not being complete, this biotransformation managed to reduce the bioavailability of ZEA, and consequently the exposure to this toxin.

The yeast *S. cerevisiae* is frequently used in human and animal nutrition, offering beneficial properties. They can be used as an additive for animal nutrition, enhancing productivity and health parameters, and also minimizing the bioavailability of mycotoxins present in the diet [22]. 

Finally, an evaluation of yeasts biotransformation ability of α-ZOL and β-ZOL was undertaken. Experiments with YPD supplemented with these ZEA modified forms were performed and culture media and cells were extracted together. The yeasts were not able to biotransform α-ZOL from culture media but they were able to biotransform 6% of the β-ZOL added to culture media. These observations strengthen the conclusion that ZEA is being subsequently metabolized into another compound by the yeasts. This metabolic effect is only possible with the use of active strains. Therefore, when living *S. cerevisiae* are added to animal feed the earnings may go beyond productivity and health performance parameters. 

## 3. Experimental Section

### 3.1. Biological Material

Three strains of *S. cerevisiae* isolated from bovine forage coded as *S. cerevisiae* LL74, LL08, and LL83 were used. Strains were deposited in the culture collection of the Research Center for Mycology and Mycotoxicology from the Federal Rural University of Rio de Janeiro (NPMM-UFRRJ) and in the National University of Río Cuarto (Córdoba, Argentina) collection center. A commercial *S. cerevisiae* strain NCYC996 (coded as CS) was used as a control strain. Yeasts were propagated at 30 °C for 2 days in falcon tubes containing 10 mL of YPD broth (1% yeast extract, 2% peptone and 2% glucose) and D.O. was determined at 600 nm and adjusted to 2.0 with sterile distilled water. Throughout the study, the strains were maintained active in YPD broth and preserved at 4 °C.

### 3.2. Adsorption of ZEA, α-ZOL and β-ZOL by S. cerevisiae Strains

Strains were tested in YPD + ZEA: YPD broth supplemented with 2 μg/mL of ZEA (Sigma-Aldrich, Sintra, Portugal). Falcon tubes with 5 mL of YPD + ZEA were inoculated in triplicate with 0.1 mL of inoculum. Negative controls were also prepared using 0.1 mL of sterile distilled water. Incubation was performed at 30 °C for 4 days. Throughout the incubation period, for each strain, triplicates were extracted and analyzed for the presence of ZEA at 0, 6, 20, 28, 48 and 72 h. Tubes were centrifuged at 10000× *g* for 10 min and the supernatants were collected. Zearalenone was determined separately in yeast pellets and supernatants as described below. This procedure was repeated for α-ZOL and β-ZOL (Sigma-Aldrich, Sintra, Portugal).

Adsorption of ZEA on yeasts cell walls was confirmed in a static gastrointestinal model. First, yeasts were cultivated in 250 mL YPD at 30 °C in an orbital shaker (150 rpm), culture broths were centrifuged at 10000× *g* for 10 min, supernatant was discarded, and pellets were frozen at −80 °C and lyophilized to obtain dried cells. The gastric simulation solution was composed by physiologic solution and enzymes: 125 mM NaCl, 7 mM KCl, 45 mM NaHCO_3_ and 3 g/L pepsin (porcine gastric mucosa, 800–2500 U/mg) at pH 3. The intestinal simulation solution was composed by physiologic solution and enzymes: 0.5% bile (*w/v*), 1 mg/mL trypsin type IX-S (13000–20000 BAEE U/mg) and 1 mg/mL α‑chymotrypsin type II (pancreas, ≥40 U/mg) at pH 6 (all from Sigma-Aldrich, Sintra, Portugal). All reaction solutions were prepared at the time of use and supplemented with 2 μg/mL of ZEA. Experiments were conducted in triplicate, by adding yeasts lyophilized powder at a concentration of 2.0 mg/mL. The concentration of 2.0 mg/mL corresponds to the recommended dose of the commercial product (2 kg/ton, with a cell count ≥ 10^6^ CFU/g). Reaction assays were incubated during 1 h at 37 °C (150 rpm). Solutions were then centrifuged at 10000× *g* for 10 min, supernatants collected and ZEA determined by HPLC-FL as described below. The percentage of ZEA adsorbed by yeast cells was calculated using Equation (1).

(1) $$\text{Adsorption }\left(\text{\%}\right)\text{=}\left[\text{1–}\frac{\text{ZEA concentration in supernatant}}{\text{ZEA concentration in positive control}}\right]\text{×100}$$

### 3.3. Biotransformation of ZEA, α-ZOL and β-ZOL by S. cerevisiae Strains

Strains were tested in YPD+ZEA as described previously. Incubation was also performed at 30 °C for 4 days. Throughout the incubation period, for each strain, triplicates were extracted and analyzed for the presence of ZEA at 0, 6, 20, 28, 48 and 96 h. Culture media were extracted together with yeast cells as described below.

The time required for 50% and 90% of ZEA degradation (DT50 and DT90, respectively) was determined by fitting a logistic model to the concentration of ZEA over time according to Equation (2).

(2) $$S=S_{0}\text{*}{\left[\frac{a_{max}}{a_{max}-a_{0}+a_{0}\text{*}e^{\left(r\text{*}t\right)}}\right]}^{\frac{a_{max}}{r}}$$

where, *S* is the percentage of ZEA present at time t, *S*_0_ is the initial percentage of ZEA present in the assay, *a*_max_ is the maximum value of the degradation constant, *a*_0_ is the initial value of the degradation constant, r is the microbial growth rate and t is the time as described in [23].

Data were fitted using a nonlinear least squares fit regression using GraphPad Prism version 6.05 (GraphPad Software, California, USA). To perform the regression analysis, ZEA data were converted to a 0% to 100% scale. *S*_0_ was constrained to 100%. The *a*_max_, *a*_0_, and *r* parameters were estimated using by fitting Equation (2) to experimental data and estimated parameters were used to calculate the DT50 and DT90 according to Equations (3) and (4), respectively.

(3) $$\text{DT}50=\frac{1}{r}\text{*}\ln\left[1-\frac{a_{max}}{a_{0}}\text{*}\left(1-2^{\frac{r}{a_{max}}}\right)\right]$$

(4) $$\text{DT}90=\;\frac{1}{r}\text{*}\ln\left[1-\frac{a_{max}}{a_{0}}\text{*}\left(1-10^{\frac{r}{a_{max}}}\right)\right]$$

For the comparison of means of quantitative variables, samples were first tested for homogeneity of variances by Levene’s test. Since samples followed this criterion, variances were analyzed by one-way ANOVA, and multiple comparisons between samples pairs were made by Bonferroni’s test. In all cases, the mean differences were significant at *p* < 0.05.

### 3.4. Extraction and Detection of Mycotoxins

Culture media with or without yeast cells and gastro-intestinal simulation solutions were extracted with an equivalent volume of acetonitrile/methanol/acetic acid (78:20:2, *v/v/v*) using vortex agitation for 1 min. Two mL samples were collected and filtered into clean amber borosilicate glass vials using syringe-fitted PP filters with 0.45 µm pores (VWR, Carnaxide, Portugal). Cell pellets were extracted with 1 mL of the same solution and 1 mL of H_2_O_d_ during an agitation period of 1 h and filtered into glass vials. The samples were preserved at 4 °C until HPLC analysis.

The HPLC system comprised a Prostar 210 pump, a Prostar 410 autosampler (all Varian, Porto, Portugal) and a FP-920 fluorescence detector (Jasco, Tokyo, Japan). The instrument and the chromatographic data were managed by a Varian 850-MIB data system interface and a Galaxie chromatography data system (all from Varian, Porto, Portugal), respectively. The chromatographic separation was performed at 30 °C on a C18 reversed-phase YMC-Pack ODS-AQ analytical column, 250 × 4.6 mm, 5 µm (YMC, Kyoto, Japan) that was fitted with a pre-column with the same stationary phase. To analyze ZEA and its derivatives [24], samples were eluted using a flow rate of 1.0 mL/min for a 23 min isocratic run with methanol/water/acetic acid (65:35:1, *v/v/v*). The injection volume was 50 µL and the fluorescence detection was performed at λ_exc_ = 236 nm, λ_em_ = 418 nm and gain = 1000. Recorded retention times for α-ZOL, β-ZOL and ZEA were approximately 12.5, 18.5, and 21.0 min, respectively. Calibration curves were prepared with α-ZOL and ZEA standards (Sigma-Aldrich, Sintra, Portugal) at concentration between 0.25 and 2.0 μg/mL. To quantify β-ZOL, a calibration curve at concentration between 2.5 and 20.0 μg/mL was prepared with standards (Sigma-Aldrich, Sintra, Portugal).

## 4. Conclusions

*S. cerevisiae* strains LL74, LL83, and LL08 isolated in this study tolerate the *in vitro* gastrointestinal conditions passage and have the ability to adsorb the toxin at a first stage, and later to metabolize it in other compounds. The main ZEA metabolite detected was the less estrogenic β-ZOL (representing about 53% of initial ZEA amount), being the more estrogenic α-ZOL present at much lower amounts (about 8%). Although at a lower level, β-ZOL is still estrogenic for what it is desirable to have a full picture on the estrogenicity of all transformation products obtained from ZEA. The simple elimination of ZEA is not enough to guarantee the safety of the feed. These isolates are promising alternatives for the development of new additives with probiotic, as well as anti-mycotoxin activity. This factor may show that new products can be developed from this modelling research. This promising development reinforces the idea that the additives of biological origin can be a viable solution in animal husbandry.
